# How effective are physical activity interventions in improving physical self-concept among children and adolescents? A systematic review and meta-analysis

**DOI:** 10.3389/fpsyg.2026.1963989

**Published:** 2026-10-16

**Authors:** Hongqiang Wang, Chaoqiong Feng, Shiwei Chen

**Affiliations:** 1 Department of Physical Education, Lanzhou University, Lanzhou, China; 2 Sports Science Research Institute, Dankook University, Cheonan, Republic of Korea; 3 School of Sport Science, Beijing Sport University, Beijing, China

**Keywords:** adolescents, children, intervention, physical activity, physical self-concept

## Abstract

**Background:**

Physical self-concept is a key indicator of physical and psychological development in children and adolescents and has been linked to participation in physical activity and overall wellbeing. Nevertheless, intervention studies examining whether structured physical activity enhances physical self-concept have yielded inconsistent findings. This review was undertaken to quantify the effectiveness of physical activity interventions and to examine factors that may influence their effects.

**Methods:**

Relevant studies were identified through systematic searches of PubMed, Web of Science, Embase, the Cochrane Library, and SPORTDiscus (via EBSCOhost) from database inception to June 10, 2026. Eligible intervention studies were quantitatively synthesized using standardized mean differences (Hedges' g) under a random-effects model. Prespecified subgroup analyses, meta-regression, leave-one-out sensitivity analyses, and publication bias assessments were also performed.

**Results:**

Ten studies involving 3,662 participants contributed to the quantitative synthesis. Overall, structured physical activity interventions were associated with greater improvements in physical self-concept than control conditions (Hedges' g = 0.51, 95% CI 0.17–0.85; *p* = 0.008), although substantial between-study heterogeneity was observed (I^2^ = 86.5%). No statistically significant differences in intervention effects were detected across geographical regions, participant types, or developmental stages. Neither intervention duration nor the proportion of female participants significantly explained between-study variability.

**Conclusion:**

Structured physical activity interventions are associated with improvements in physical self-concept among children and adolescents. These findings suggest that structured physical activity may be a useful strategy for promoting positive physical self-concept during childhood and adolescence.

## 1 Background

Physical self-concept is widely recognized as an important psychological determinant of healthy development in children and adolescents. It reflects how individuals perceive their physical competence, fitness, strength, appearance, and overall physical worth, and these perceptions influence motivation, physical activity participation, body image, and psychological wellbeing (Shavelson et al., 1976; Fox and Corbin, 1989; Lindwall et al., 2011; Fernández-Bustos et al., 2019). Childhood and adolescence represent particularly sensitive developmental periods because rapid physical maturation, changing body composition, and increasing peer comparison continuously shape adolescents's evaluations of their physical selves (Voelker et al., 2015). Consequently, promoting a positive physical self-concept has become an important objective of interventions designed to support healthy youth development.

Among the factors associated with physical self-concept, physical activity has received particular attention because it is both modifiable and readily implemented in schools and community settings. Beyond improving physical fitness, participation in physical activity provides repeated opportunities to acquire motor skills, experience mastery, and receive positive social feedback, all of which may strengthen perceptions of physical competence and physical appearance (Burton et al., 2023). Recent evidence further highlights the developmental relevance of physical competence and perceived physical capability. Motor competence during childhood may vary according to individual characteristics such as sex and body mass index (Kumar et al., 2025), while the ability to accurately perceive physical effort also develops during pre-adolescence (Choudhary et al., 2024). In addition, collaborative and game-based approaches in physical education may provide meaningful opportunities for movement-skill development and mastery experiences (Saha et al., 2026). Consistent with this perspective, observational evidence indicates that children and adolescents who engage in higher levels of physical activity generally report more positive physical self-concept than their less active peers (Babic et al., 2014). These findings provide a theoretical rationale for using structured physical activity interventions to enhance physical self-concept.

A growing number of intervention studies have examined whether increasing physical activity can improve physical self-concept in children and adolescents. These interventions vary considerably, including school-based physical education, aerobic exercise, resistance training, dance, yoga, exergaming, adventure education, and structured after-school physical activity programs (Zamorano-García et al., 2023). Some studies have reported improvements in body attractiveness, physical self-worth, and domain-specific physical self-concept following structured physical activity programs (Burgess et al., 2006; Schmidt et al., 2013). However, other studies have found little or no improvement despite increases in physical fitness or prolonged participation in physical activity programs (Mayorga-Vega et al., 2012; Christiansen et al., 2018). These inconsistent findings suggest that intervention effectiveness may depend on factors such as participant characteristics, intervention modality, program duration and intensity, implementation quality, and the dimensions of physical self-concept being assessed.

An earlier meta-analysis by Zamorano-García et al. (2023) provided important evidence on the effects of physical activity-based interventions on physical self-perceptions in children and adolescents. However, that review adopted a relatively broad outcome framework, synthesizing overall physical self-concept alongside a range of related but distinct constructs, including physical appearance, sport competence, physical fitness, and global self-concept. Consequently, the evidence synthesized in that review extended beyond outcomes directly representing overall physical self-concept. In contrast, the present review specifically focused on outcomes that directly measured overall physical self-concept and did not use related physical self-perception constructs as substitutes for the overall construct. This more focused outcome definition allows the present meta-analysis to provide a conceptually more specific estimate of the effect of structured physical activity interventions on physical self-concept itself. In addition, the previous review searched the literature up to February 2020, whereas the present review incorporates evidence available through June 2026 and further examines potential sources of between-study variability through subgroup and meta-regression analyses.

Accordingly, this review quantitatively synthesized the available evidence to estimate the overall effect of physical activity interventions on physical self-concept in children and adolescents and to determine whether intervention effects varied according to participant- and study-related characteristics. By providing an updated and more focused synthesis of physical self-concept outcomes, this review seeks to clarify the magnitude and consistency of intervention effects and to identify potential sources of between-study variability, thereby informing the design of future physical activity interventions aimed at supporting positive physical self-development.

## 2 Methods

### 2.1 Literature search and selection

This review was designed and reported in accordance with the PRISMA 2020 statement (Page et al., 2021). Registration No. CRD420261474718.

Relevant studies were identified through systematic searches of PubMed, Web of Science, Embase, the Cochrane Library, and SPORTDiscus via EBSCOhost from database inception to June 10, 2026. The search strategy used free-text terms and relevant keywords related to physical activity, exercise, physical self-concept, physical self-perception, children, and adolescents. Detailed search strategies for all databases are available in Supplementary File 1.

Following database searching, duplicate records were removed prior to title and abstract screening. The full texts of potentially eligible articles were subsequently assessed against the predefined eligibility criteria. Reference lists of the included studies and related review articles were additionally examined to identify any eligible studies not retrieved through the electronic searches. The literature search and study selection were completed independently by two investigators (H.W. and C.F.). Any discrepancies were first discussed between the two reviewers, and unresolved disagreements were settled by the corresponding author (S.C.).

### 2.2 Inclusion and exclusion criteria

Studies were considered eligible if they met the following criteria: (1) included children or adolescents with a reported sample mean age below 18 years; (2) evaluated a structured physical activity or exercise intervention; (3) included a comparison group receiving usual practice, no intervention, or another non-equivalent control condition; (4) assessed overall/global physical self-concept or physical self-worth using a validated instrument; (5) reported sufficient quantitative data to calculate standardized effect sizes; and (6) employed an intervention study design.

Eligible interventions were required to involve a structured physical activity or exercise component in which the intervention group received a greater or substantively different physical activity or exercise exposure than the comparison group. Studies in which both groups received essentially the same physical activity programme and the experimental manipulation consisted primarily of a non-physical component, such as self-assessment or educational strategies, were not considered eligible physical activity interventions. Physical education-based interventions were not excluded solely because they were delivered within a school or physical education setting; rather, eligibility depended on whether physical activity or exercise constituted the substantive intervention contrast between groups.

Regarding outcome measures, eligible outcomes included overall/global physical self-concept and physical self-worth assessed using validated instruments. Studies reporting only specific physical self-perception dimensions, such as perceived strength, endurance, coordination, sport competence, physical condition, or body attractiveness, were excluded. General self-concept, global self-esteem, and other outcomes that did not specifically reflect the physical self were also excluded. When a multidimensional physical self-concept or physical self-perception instrument was used, eligibility was determined according to the specific outcome extracted rather than the instrument as a whole.

Studies were excluded if they: (1) were observational studies, case reports, conference abstracts, reviews, dissertations, or study protocols; (2) did not include an eligible comparison group; (3) did not meet the intervention or outcome criteria described above; (4) lacked sufficient data for effect size calculation after attempts to obtain additional information from the study authors; (5) had a reported sample mean age of 18 years or older; or (6) were not published in English.

### 2.3 Data extraction and coding process

Two investigators (H.W. and C.F.) independently collected study data using a standardized extraction template developed for this review. Differences between reviewers were resolved through discussion, and any remaining disagreements were settled by the corresponding author (S.C.).

Information collected from each study covered publication details (first author, publication year, and country), participant characteristics (sample size, age, sex, population type, and developmental stage), intervention characteristics (intervention and control conditions together with intervention duration), outcome measures, and the statistical data required for effect size estimation. Specifically, sample sizes together with baseline and post-intervention means and standard deviations were extracted separately for the intervention and comparison groups.

To facilitate subgroup analyses, studies were coded according to geographical region, participant type, and developmental stage. Countries were classified into Europe, North America, or the Asia–Pacific region, with Australia categorized as part of the Asia–Pacific region. Participant type was coded as either general populations or special populations. General populations included participants without predefined health- or self-concept-related characteristics, whereas special populations included participants with asthma, overweight or obesity, low physical activity, low physical self-perceptions, body-image dissatisfaction, or other characteristics explicitly reported in the original studies that could influence physical self-concept. For the developmental-stage subgroup analysis, studies were classified as involving children when the reported mean age was ≤ 12 years and adolescents when the reported mean age was >12 years. This classification was used solely for subgroup analysis and was distinct from the age criterion used to determine study eligibility. This classification was used solely for subgroup analysis and was distinct from the age criterion used to determine study eligibility when the exact age range was not reported, eligibility was determined based on the reported mean age and the authors' description of the study population.

### 2.4 Risk of bias and publication bias assessment and certainty of evidence

The risk of bias of the included studies was assessed using design-appropriate tools. Randomized studies were evaluated using the revised Cochrane Risk of Bias tool (RoB 2) (Sterne et al., 2019), whereas non-randomized intervention studies were assessed using the Risk Of Bias In Non-randomized Studies of Interventions (ROBINS-I) tool (Sterne et al., 2016). Differences in assessment were discussed between the reviewers, and unresolved cases were decided by the corresponding author (S.C.).

Publication bias was evaluated using both visual inspection of funnel plots and Egger's regression test (Egger et al., 1997). A two-sided *p*-value below 0.05 was interpreted as indicating possible publication bias (Sterne et al., 2011).

The certainty of evidence for the primary outcome was assessed using the Grading of Recommendations Assessment, Development and Evaluation (GRADE) approach, considering risk of bias, inconsistency, indirectness, imprecision, and publication bias. The overall certainty of evidence was classified as high, moderate, low, or very low (Guyatt et al., 2008).

### 2.5 Statistical analysis

The quantitative synthesis was performed by pooling intervention effects on physical self-concept across eligible studies. Standardized mean differences were estimated using Hedges' g and corresponding 95% confidence intervals (CIs), allowing results obtained from different physical self-concept instruments to be synthesized on a common scale (Turner and Bernard, 2006).

Effect sizes were calculated from between-group differences in pre–post change scores. Mean change was defined as the post-intervention mean minus the baseline mean. When change-score means and standard deviations were reported directly, these values were used. When change-score means were not reported, they were calculated as the post-intervention mean minus the baseline mean. When change-score standard deviations were unavailable, they were estimated from the baseline and post-intervention standard deviations using the following formula:


$$\begin{array}{l} \text{SDchange}=\text{√}\left(SDpre^{2}+SDpost^{2}-2r\times SDpre\times SDpost\right). \end{array}$$

A pre–post correlation coefficient of r = 0.50 was used as a moderate primary assumption for studies requiring estimation of the change-score standard deviation when the within-participant correlation was unavailable. Sensitivity analyses were conducted using r = 0.20 and r = 0.80 to examine the influence of lower and higher correlation assumptions. Standardized mean differences were corrected for small-sample bias and expressed as Hedges' g, with positive values indicating greater improvement in the intervention group. The correlation-coefficient and cluster/site-level sensitivity analyses were conducted separately because they addressed distinct sources of methodological uncertainty.

To maintain statistical independence, each study contributed only one effect estimate to the primary meta-analysis. When multiple physical self-concept outcomes were reported, the outcome most directly representing the primary physical self-concept construct evaluated in the study was selected. When multiple eligible intervention arms shared a common control group, one intervention–control comparison representing the primary intervention was included to avoid repeated use of the same control participants. When outcomes were reported at multiple time points, data collected immediately after completion of the intervention were used. For the crossover study, only first-period data collected before crossover were included to avoid within-participant dependence and potential carryover effects.

A random-effects model was adopted because methodological and clinical differences among the included studies were expected (Borenstein et al., 2010). The between-study variance (τ^2^) was estimated using restricted maximum likelihood (REML), while confidence intervals were derived using the Hartung–Knapp adjustment, which is recommended for meta-analyses including relatively few studies (Viechtbauer, 2005; Hartung and Knapp, 2001).

Heterogeneity across studies was examined using Cochran's Q test and further quantified by the I^2^ statistic (Thompson and Higgins, 2002). Subgroup analyses were performed based on geographical region, participant type, and developmental stage. Meta-regression analyses were further undertaken to investigate whether intervention duration and the proportion of female participants were associated with the observed intervention effects.

Robustness of the overall findings was evaluated using a leave-one-out procedure, whereby each study was removed sequentially and the pooled estimate recalculated (Viechtbauer and Cheung, 2010). For cluster-allocated studies, the original reports were examined to determine whether clustering had been accounted for in the statistical analysis. For studies with cluster- or site-level allocation, we examined whether the original analyses accounted for within-cluster dependence and whether sufficient information was available to implement an appropriate adjustment in the present meta-analysis. Where effect sizes were calculated from group-level means, standard deviations, and sample sizes, conventional SMD sampling variances were used. An additional design-effect correction was not applied when an appropriate outcome-specific intracluster correlation coefficient (ICC) and/or sufficient cluster-level information was unavailable, because imposing an arbitrary ICC could introduce additional assumptions. When appropriate adjustment for clustering could not be confirmed, an additional sensitivity analysis was conducted by excluding these studies to assess the robustness of the pooled estimate. Possible small-study effects were examined through funnel plot inspection and Egger's regression test.

Statistical analyses were carried out in R (version 4.4.0) with the meta and metafor packages (Viechtbauer, 2010). A two-sided *p*-value below 0.05 was considered statistically significant.

## 3 Results

### 3.1 Search results and study characteristics

The database search yielded 2,455 records: 286 from PubMed, 233 from Web of Science, 413 from Embase, 1,193 from the Cochrane Library, and 330 from EBSCO. Removal of 479 duplicates left 1,976 records for title and abstract screening, after which 1,920 records were excluded. Full-text evaluation was conducted for 56 articles retrieved from the database search, and a further 30 records were located through website searches and citation tracking. At the eligibility stage, 20 reports were excluded because of incompatible outcome measures, 11 because of insufficient data, and 20 because of ineligible study designs. The final evidence base comprised 10 studies included in both the systematic review and quantitative synthesis (Röglin et al., 2024; Mayorga-Vega et al., 2012; Lindwall and Lindgren, 2005; Latorre-Román et al., 2015; Schranz et al., 2014; Annesi et al., 2008; Christiansen et al., 2018; Annesi, 2005, 2006; Burgess et al., 2006). The study selection process is presented in Figure 1.

**Figure 1:**
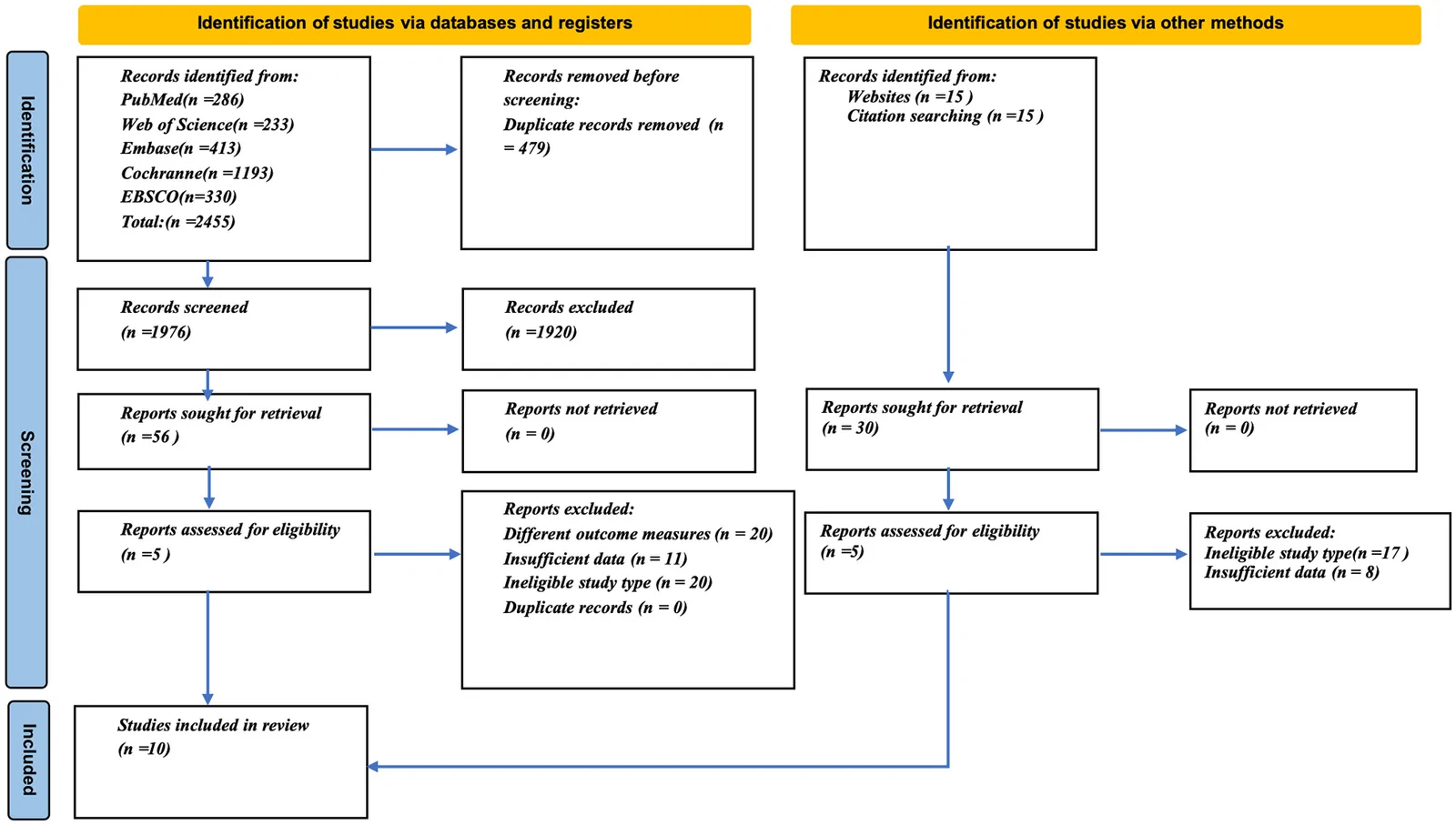
PRISMA 2020 flow diagram summarizing the study selection process.

The evidence base comprised 10 eligible studies, with reported study sample sizes ranging from 50 to 2,797 participants. Of these, 3,662 participants contributed to the quantitative synthesis, including 1,758 participants in the intervention groups and 1,904 in the control groups. Publication years ranged from 2005 to 2024, and the studies were conducted across seven countries. Six studies were conducted in Europe, three in North America, and one in the Asia–Pacific region. Six studies recruited participants from general populations, whereas four focused on populations with specific characteristics, including physically inactive adolescent girls, children with asthma, overweight or obese adolescent boys, and adolescent girls with low physical self-perceptions. Interventions included school-based physical activity and exergaming programmes, aerobic exercise, resistance training, dance, and other structured exercise programmes, with durations ranging from 6 weeks to 9 months. Eligible outcomes included overall/global physical self-concept and physical self-worth, assessed using validated instruments, including the Physical Self-Perception Profile and its variants, the Physical Self-Description Questionnaire, the Physical Self-Concept Questionnaire, and the Tennessee Self-Concept Scale-2 Child Form. Detailed study characteristics are presented in Table 1.

**Table 1:** Characteristics of included literature.

References	Country	N (total sample)	Outcome measure	Intervention programme	Intervention schedule	Participant type	Female (%)	Age (years)
Röglin et al. (2024)	Germany	58	Physical self-concept total score (PSC)	School-based exergaming programme	12 weeks; 2 sessions/week; 15–20 min/session	Grade 5–6 primary school students	46.6	10.4 ± 0.5
Mayorga-Vega et al. (2012)	Spain	75	Global physical self-concept (PSDQ)	Circuit-based physical fitness programme	8 weeks; 2 sessions/week; 50 min/session	Grade 6 primary school students	45.3	11.1 ± 0.4
Lindwall and Lindgren (2005)	Sweden	110	Physical self-worth (PSPP)	Exercise intervention programme	6 months; 2 sessions/week; 60 min/session	Non-physically active adolescent girls	100	16.35 ± 1.56
Latorre-Román et al. (2015)	Spain	105	General physical self-concept (CAF)	Indoor physical activity programme	12 weeks; 3 sessions/week; 60 min/session	Children with asthma (10–14 years)	39	11.53 ± 1.20
Schranz et al. (2014)	Australia	56	Physical self-worth (PSW)	Supervised resistance training programme	6 months; 3 sessions/week; 75 min/session	Overweight/obese adolescent boys (13–17 years)	0	15.0 ± 1.5
Annesi et al. (2008)	USA	269	Physical self-concept (TSCS-2 Child Form)	Youth Fit For Life programme	12 weeks; 3 sessions/week; 45 min/session	African American preadolescents	59	10.6 ± 1.1
Christiansen et al. (2018)	Denmark	2797	Physical self-worth (C-PSPP)	Multi-component school-based physical activity programme	9 months	Grade 4–6 primary school students	48.9	10–13
Annesi (2005)	USA	92	Physical self-concept (TSCS-2 Child Form)	After-school physical activity programme	12 weeks; 3 sessions/week; 45 min/session	Preadolescents (9–12 years)	50	10.7 ± 1.0
Annesi (2006)	USA	165	Physical self-concept (TSCS-2 Child Form)	Youth Fit For Life programme	12 weeks; 3 sessions/week; 45 min/session	Preadolescents (9–12 years)	64	10.8 ± 1.1
Burgess et al. (2006)	UK	50	Physical self-worth (CY-PSPP)	Aerobic dance programme	6 weeks; 2 sessions/week; 50 min/session	Adolescent girls with low physical self-perceptions	100	13.5 ± 0.3

### 3.2 Risk of bias and certainty of evidence

Risk of bias was assessed using design-appropriate tools according to study design. Among the six randomized studies evaluated using RoB 2, all six were judged to raise some concerns, with no study judged to be at high overall risk of bias. The most frequent concerns related to measurement of the outcome, with additional concerns regarding deviations from intended interventions and selection of the reported result in one study. Among the four non-randomized intervention studies assessed using ROBINS-I, all four were judged to have a moderate overall risk of bias. The principal concerns among the non-randomized studies related to potential confounding, participant selection, missing outcome data, and selection of the reported result. No study was judged to have a serious or critical overall risk of bias. Detailed risk-of-bias assessments are presented in Figure 2.

**Figure 2:**
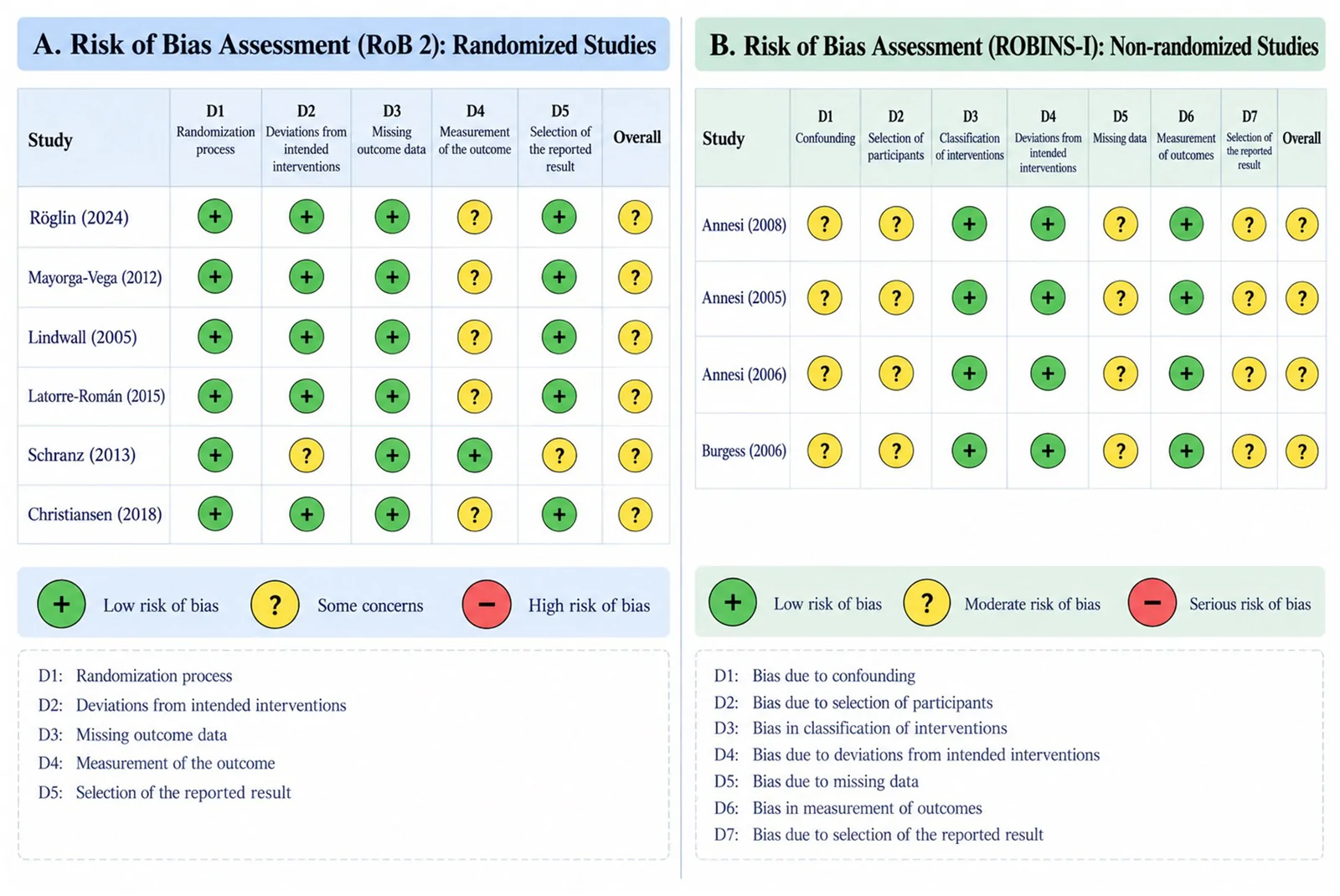
Risk-of-bias assessment of the included studies using the revised Cochrane risk of bias tool (RoB 2) for randomized studies and the risk of bias in non-randomized studies of interventions (ROBINS-I) tool for non-randomized studies.

The certainty of evidence for the primary outcome was rated as low. No additional downgrading was applied for risk of bias because, although all randomized studies were rated as having some concerns and all non-randomized studies were rated as having moderate risk of bias, none was rated as having high, serious, or critical risk of bias. The evidence was downgraded one level for inconsistency because substantial between-study heterogeneity was present (I^2^ = 86.5%). No downgrading was applied for indirectness because the included populations, interventions, and outcomes were directly relevant to the review question. No downgrading was applied for imprecision because the confidence interval around the pooled estimate did not cross the null, although uncertainty remained regarding the magnitude of the effect. The evidence was downgraded one level for concerns regarding publication bias/small-study effects because funnel-plot asymmetry was observed and Egger's regression test was statistically significant. Given the small number of included studies and substantial heterogeneity, however, the observed asymmetry was not considered definitive evidence of publication bias. Overall, these considerations resulted in a low certainty rating for the pooled evidence.

### 3.3 Publication bias and sensitivity analysis

Visual inspection of the funnel plot suggested some asymmetry (Figure 3). Consistent with this observation, Egger's regression test indicated statistically significant funnel plot asymmetry (z = 2.42, *p* = 0.016), suggesting the presence of small-study effects. However, these findings should be interpreted cautiously because only 10 studies were included and substantial between-study heterogeneity was present. Publication bias therefore cannot be excluded, and the magnitude of the pooled effect may have been influenced by small-study effects, although the observed asymmetry may also reflect heterogeneity or methodological differences among studies.

**Figure 3:**
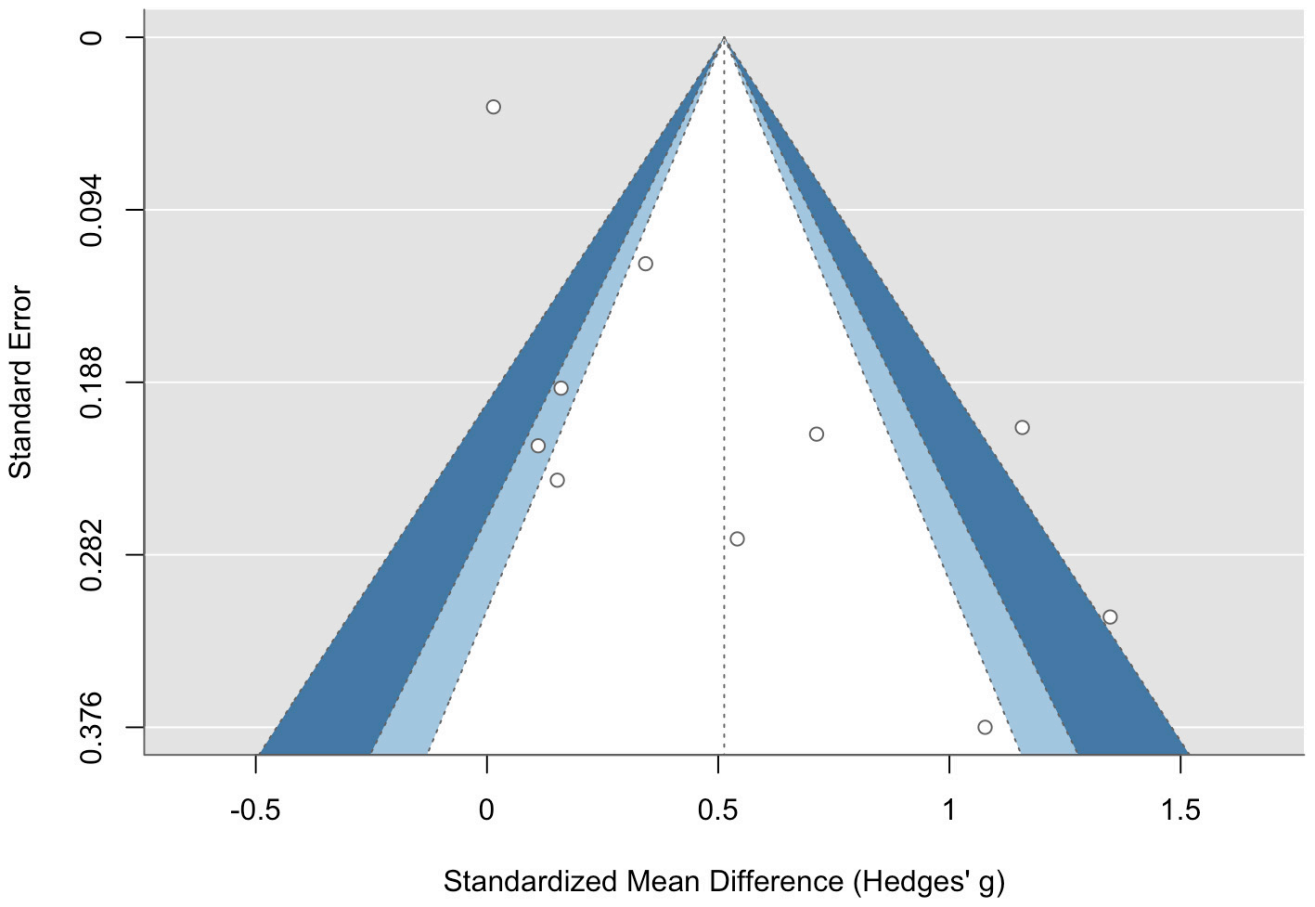
Funnel plot assessing publication bias.

Sensitivity analyses supported the robustness of the pooled effect. First, the leave-one-out analysis showed that sequentially removing individual studies resulted in only modest changes in the pooled effect estimate (Hedges' g range: 0.43–0.58) and between-study heterogeneity (I^2^ range: 73.4%−88.0%). Across all iterations, the direction of the effect remained unchanged and the pooled estimates remained statistically significant, indicating that no single study exerted an undue influence on the overall finding. Figure 4 summarizes the results.

**Figure 4:**
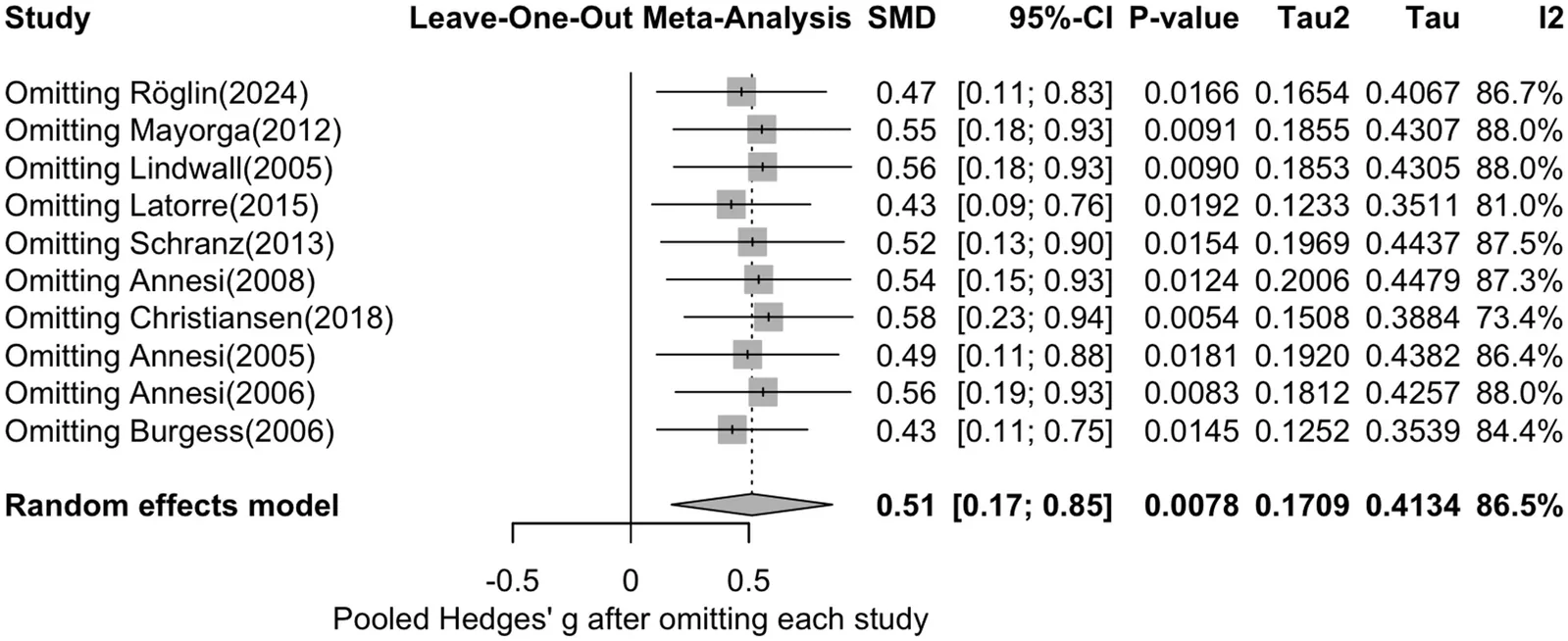
Leave-one-out sensitivity analysis of the effect of physical activity interventions on physical self-concept.

Second, because the standard deviations of change scores were estimated using an assumed pre–post correlation coefficient when directly reported change-score standard deviations were unavailable, additional sensitivity analyses were conducted using alternative correlation coefficients of r = 0.20 and r = 0.80, in addition to the primary assumption of r = 0.50. The pooled effects were g = 0.44 (95% CI: 0.14–0.74) for r = 0.20, g = 0.51 (95% CI: 0.17–0.85) for r = 0.50, and g = 0.69 (95% CI: 0.23–1.15) for r = 0.80. Although the direction and statistical significance of the pooled effect remained consistent across the assumed correlation coefficients, the magnitude of the estimated effect varied, indicating some sensitivity of the effect size to the assumed pre–post correlation.

Finally, an additional sensitivity analysis excluded four studies for which appropriate adjustment for cluster- or site-level dependence could not be implemented. The resulting pooled effect remained positive and statistically significant (Hedges' g = 0.66, 95% CI: 0.06–1.27; *p* = 0.037). Although the overall direction of the finding was not materially altered by the exclusion of these studies, the wider confidence interval indicated reduced precision and greater uncertainty in the effect estimate.

### 3.4 Main effect results

The quantitative synthesis included 10 studies comprising 3,662 participants, with 1,758 participants in the intervention groups and 1,904 in the control groups. Under the random-effects model, structured physical activity interventions were associated with greater improvements in physical self-concept and physical self-worth than control conditions (Hedges' g = 0.51, 95% CI: 0.17–0.85, *p* = 0.008). However, substantial between-study heterogeneity was observed (I^2^ = 86.5%, τ^2^ = 0.1709; Q = 66.51, *p* < 0.001), indicating considerable variability in effect sizes across studies. Therefore, the pooled estimate should be interpreted as an average effect across heterogeneous interventions and study populations rather than as evidence of a uniform intervention effect. Figure 5 summarizes the results.

**Figure 5:**
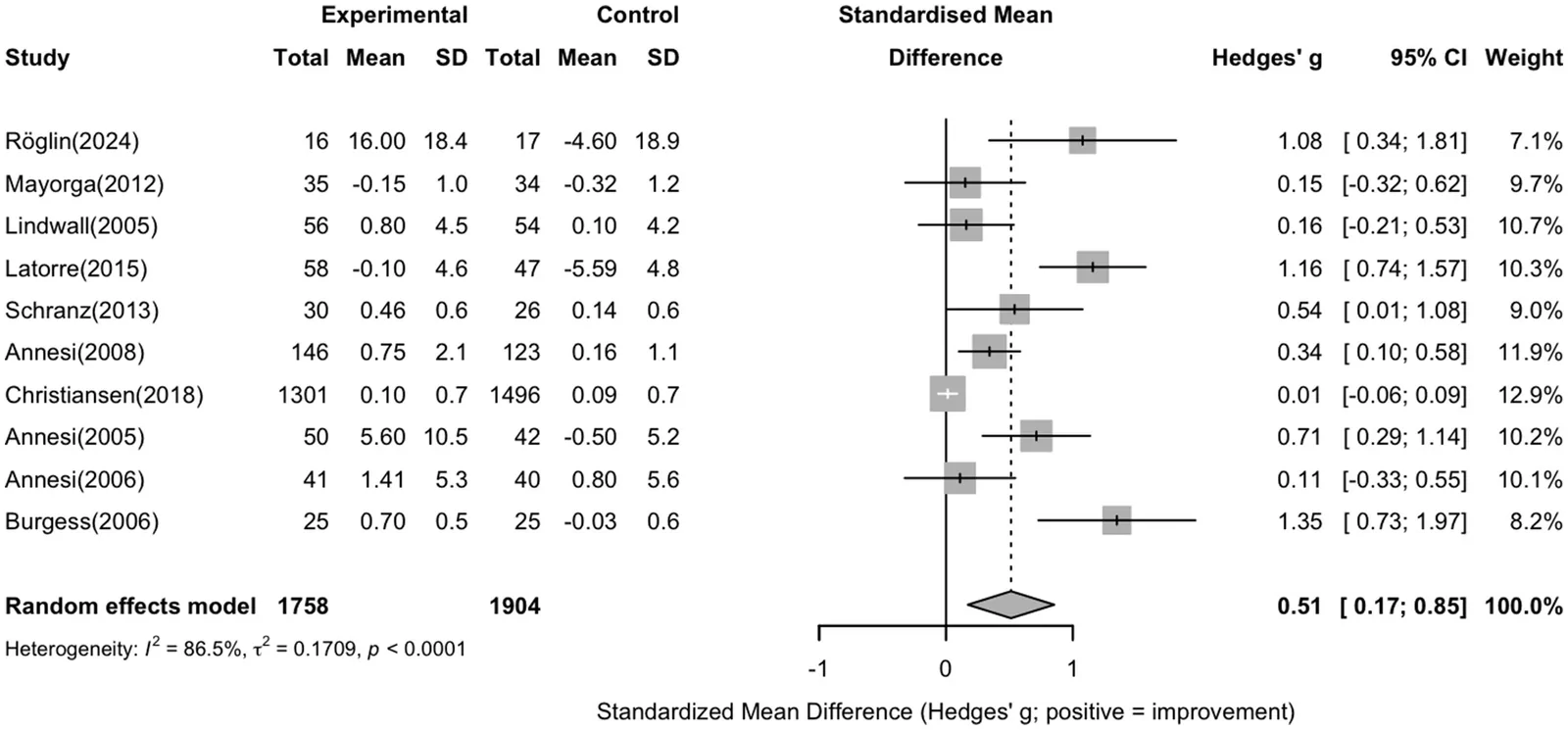
Forest plot showing the overall effect of physical activity interventions on physical self-concept.

#### 3.4.1 Subgroup analysis by geographical region

Regional differences in intervention effects were explored through subgroup analysis. Studies conducted in Europe (k = 6) showed a positive pooled effect (Hedges' g = 0.61, 95% CI: −0.03 to 1.24; I^2^ = 90.4%). A positive pooled estimate was also observed in North America (k = 3) (Hedges' g = 0.38, 95% CI: −0.30 to 1.06; I^2^ = 49.0%). However, the confidence intervals for both regional estimates included the null value. No statistically significant evidence of a difference in intervention effects between Europe and North America was detected (Q = 0.58, *p* = 0.448). Given the small number of studies within the regional subgroups and the substantial heterogeneity among European studies, these subgroup findings should be interpreted cautiously. Figure 6 summarizes the results. Because only one eligible study was conducted in the Asia–Pacific region, this region was not analyzed as a separate subgroup.

**Figure 6:**
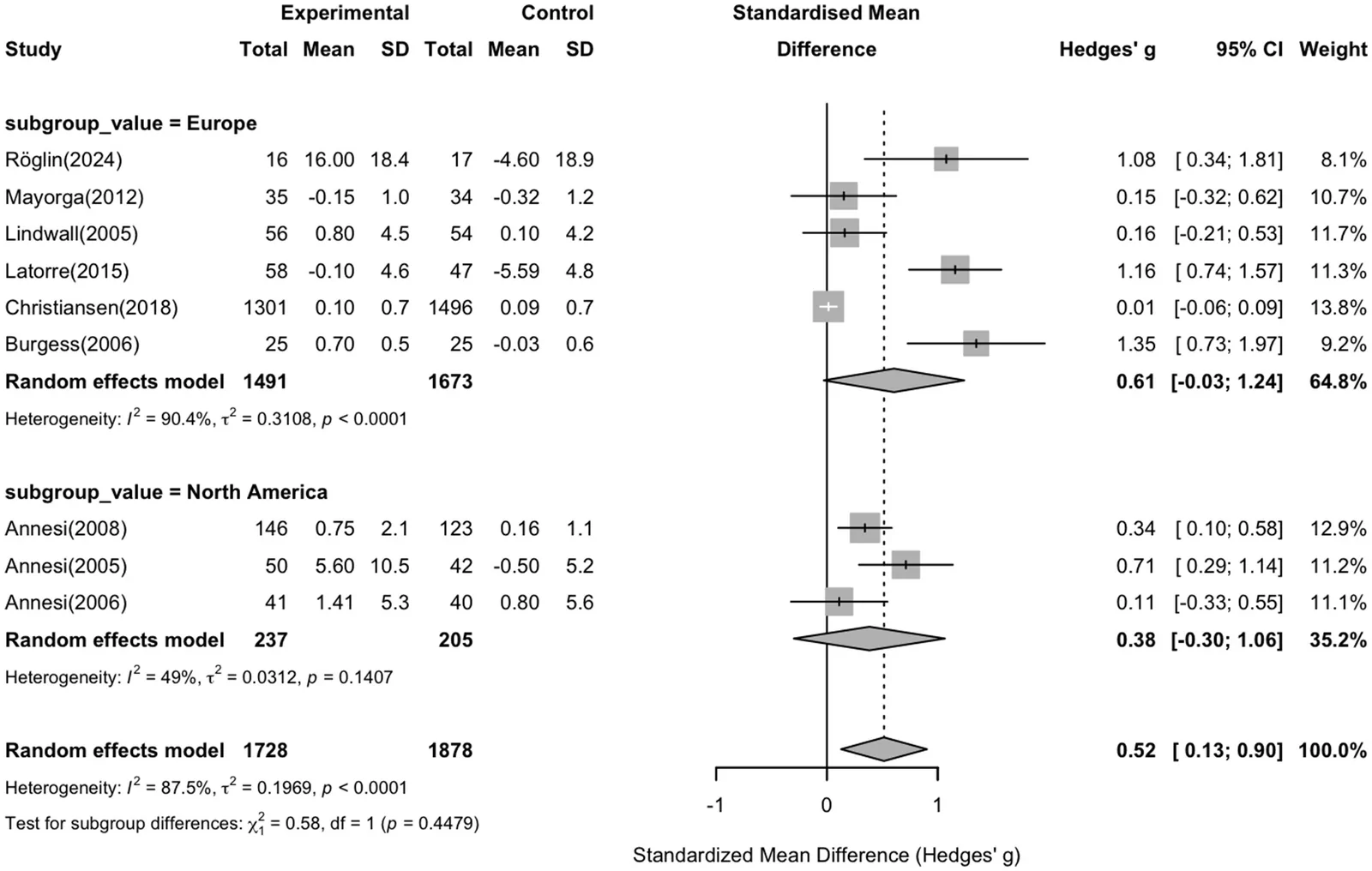
Subgroup analysis according to geographical region.

#### 3.4.2 Subgroup analysis by population type

Intervention effects were further examined according to participant type. Among general populations (k = 6), structured physical activity interventions showed a positive pooled effect on physical self-concept (Hedges' g = 0.32, 95% CI: −0.06 to 0.70), with substantial heterogeneity (I^2^= 78.5%). A larger positive pooled effect was observed among special populations (k = 4) (Hedges' g = 0.78, 95% CI: −0.10 to 1.66), although heterogeneity was considerable (I^2^= 82.5%). The confidence intervals for both subgroup estimates included the null value. No statistically significant evidence of a difference in intervention effects between participant-type subgroups was detected (Q = 2.15, *p* = 0.142). Given the relatively small number of studies, particularly in the special-population subgroup, the wide confidence intervals, and the substantial within-subgroup heterogeneity, these findings should be interpreted cautiously. Figure 7 summarizes the results.

**Figure 7:**
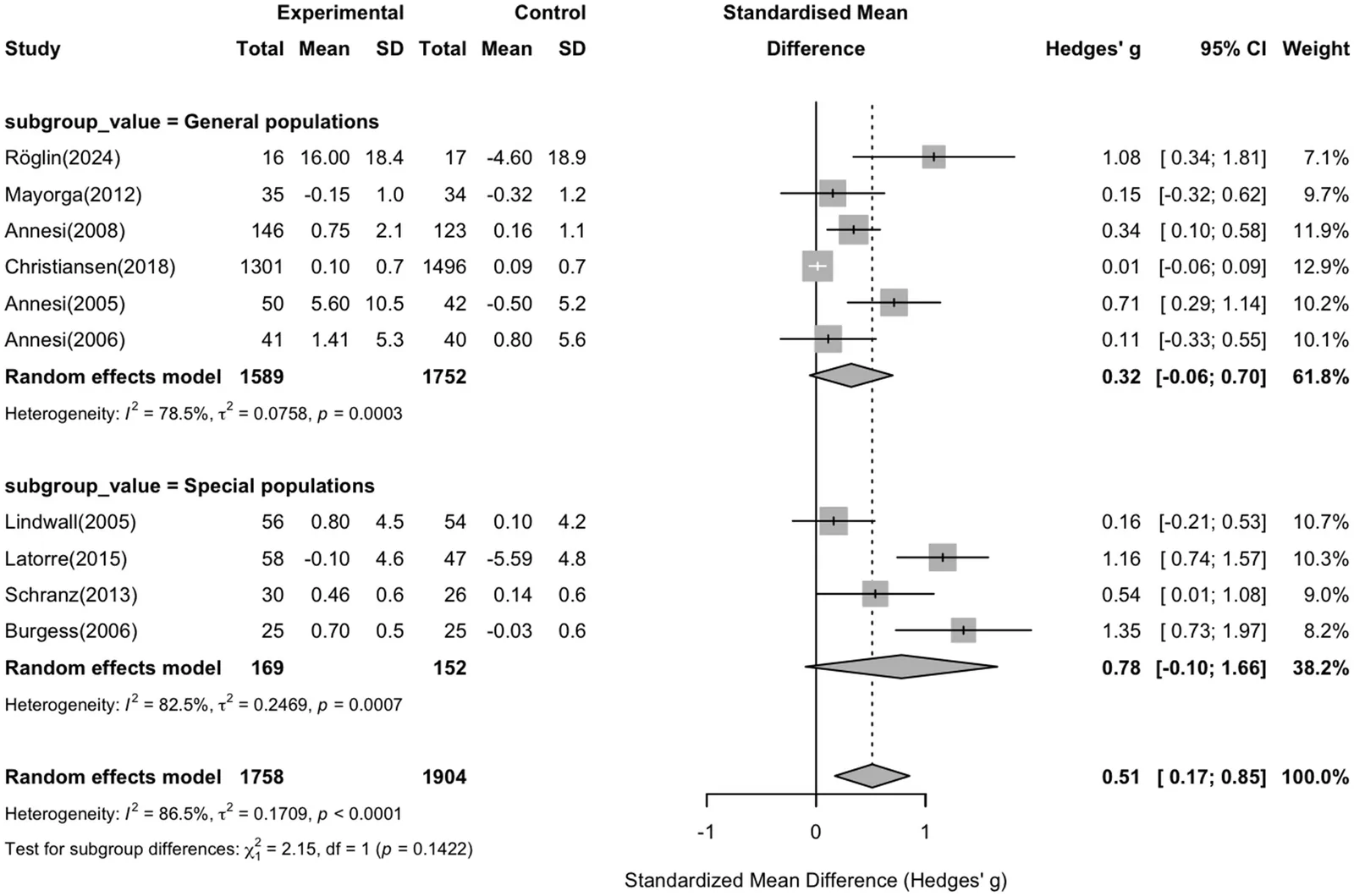
Subgroup analysis according to participant type.

#### 3.4.3 Subgroup analysis by developmental stage

Subgroup analyses were also performed according to developmental stage. Among children (k = 7), structured physical activity interventions showed a positive pooled effect on physical self-concept (Hedges' g = 0.47, 95% CI: 0.04 to 0.89), although substantial heterogeneity was observed (I^2^ = 87.7%). A positive pooled effect was also observed among adolescents (k = 3) (Hedges' g = 0.65, 95% CI: −0.84 to 2.14), with considerable heterogeneity (I^2^ = 80.8%). The confidence interval for the adolescent subgroup included the null value. There was no statistically significant evidence of a difference in intervention effects between developmental-stage subgroups (Q = 0.22, *p* = 0.638). Given the limited number of studies within each subgroup, the wide confidence intervals, and the substantial within-subgroup heterogeneity, these findings should be interpreted cautiously. Figure 8 summarizes the results.

**Figure 8:**
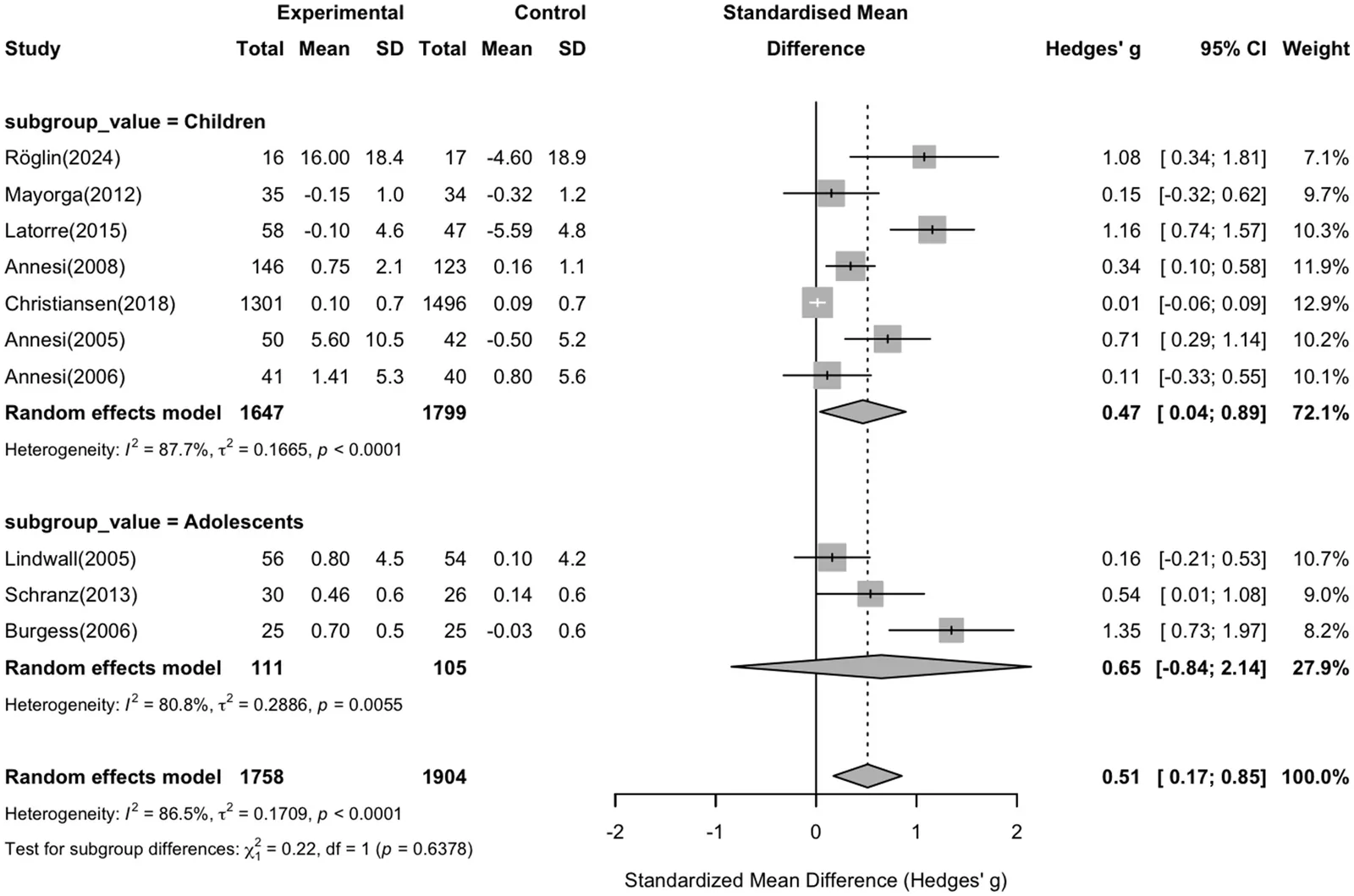
Subgroup analysis according to developmental stage.

#### 3.4.4 Meta-regression analysis

Meta-regression analyses based on random-effects models were conducted to evaluate whether intervention duration and the proportion of female participants accounted for variability in intervention effects on physical self-concept. Given the limited number of included studies (k = 10), these analyses were considered exploratory.

No statistically significant evidence was found that intervention duration explained variation in intervention effects (β = −0.023, 95% CI: −0.047 to 0.002, *p* = 0.068). Intervention duration accounted for 27.6% of the between-study heterogeneity (R^2^ = 27.6%); however, substantial residual heterogeneity remained (I^2^ = 73.6%; QE(8) = 27.25, *p* < 0.001). The corresponding regression plot is shown in Figure 9.

**Figure 9:**
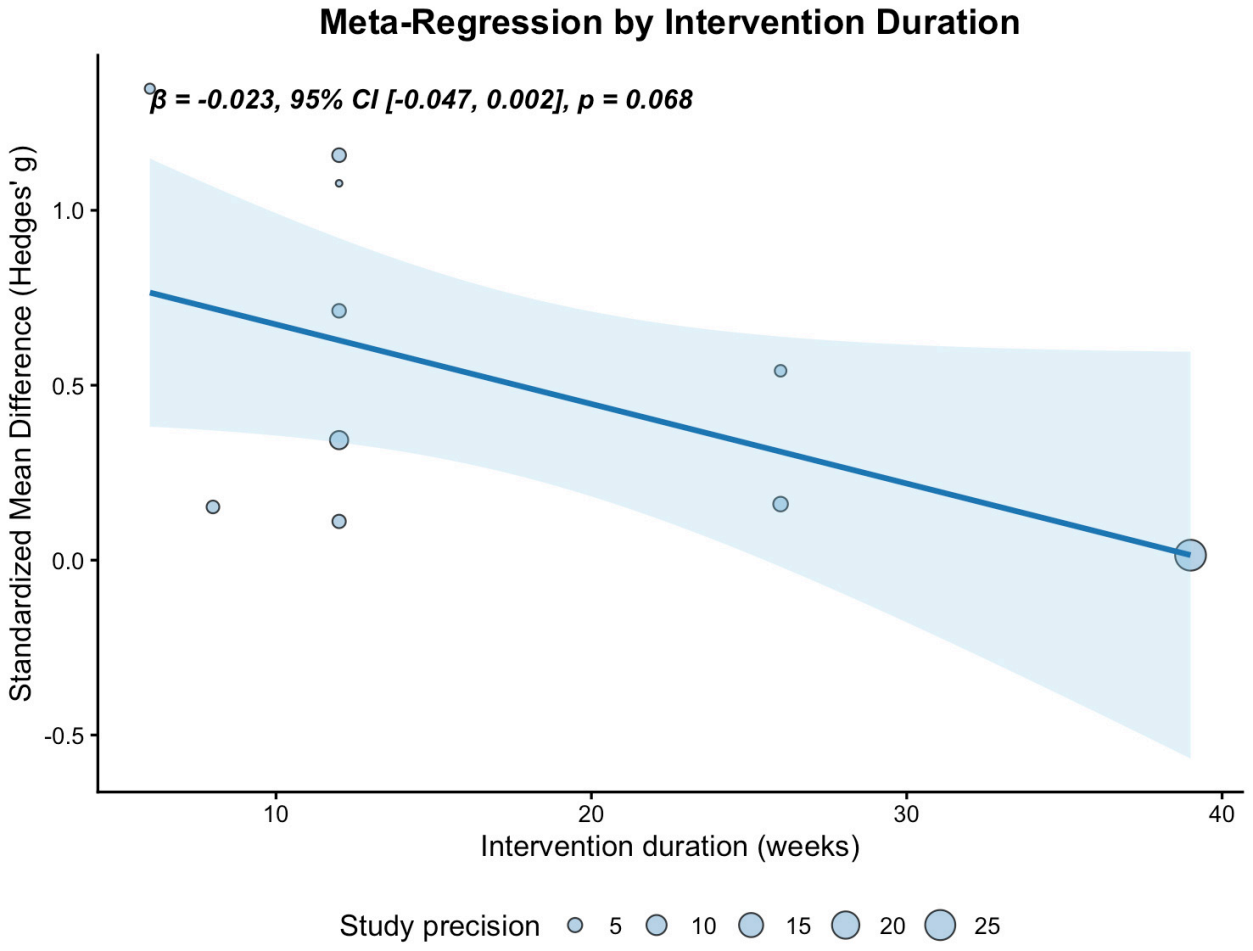
Meta-regression analysis according to intervention duration.

No statistically significant evidence was found that the proportion of female participants explained variation in intervention effects (β = 0.0001, 95% CI: −0.011 to 0.012, *p* = 0.984). The proportion of female participants accounted for 0.0% of the between-study heterogeneity (R^2^ = 0.0%); however, substantial residual heterogeneity remained (I^2^ = 88.7%; QE(8) = 65.79, *p* < 0.001). The corresponding regression plot is shown in Figure 10. Given the small number of studies (k = 10), these meta-regression analyses had limited statistical power and the estimates should be interpreted cautiously; the absence of statistically significant associations should not be interpreted as evidence that moderation is absent.

**Figure 10:**
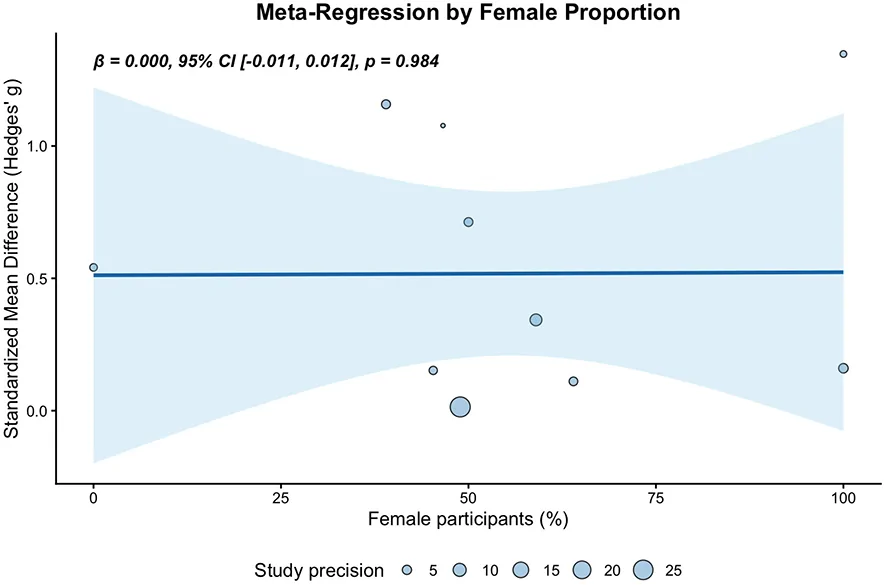
Meta-regression analysis according to the proportion of female participants.

## 4 Discussion

The present meta-analysis found that physical activity interventions were associated with greater improvements in physical self-concept in children and adolescents. Compared with participants in the control conditions, those receiving structured physical activity interventions generally reported more favorable perceptions of their physical abilities and overall physical selves. These findings suggest potential benefits of physical activity interventions for promoting positive physical self-concept during childhood and adolescence.

The present findings are consistent with previous intervention studies reporting improvements in multiple dimensions of physical self-concept following structured physical activity. Improvements have been observed in sport competence, physical condition, and physical self-worth among previously inactive adolescent girls, in overall physical self-concept among children with asthma participating in structured indoor exercise programmes, and in physical self-worth and perceived physical condition among adolescent boys following school-based interventions (Lindwall and Lindgren, 2005; Latorre-Román et al., 2015; Morgan et al., 2012). Collectively, these studies support the present findings and suggest that the psychological benefits of physical activity may extend across different populations and intervention settings.

Several mechanisms may explain the positive association observed between physical activity interventions and physical self-concept. Physical self-concept develops through repeated experiences of evaluating one's physical competence, fitness, and bodily capabilities. As children and adolescents participate in structured physical activity, improvements in physical fitness, motor skills, and task performance provide tangible evidence of personal progress, which may strengthen perceptions of physical competence and physical condition (Schneider et al., 2008). Evidence from structured mind–body exercise further suggests that physical activity may produce multidimensional benefits encompassing both physical and psychological outcomes (Choudhary et al., 2026), supporting the broader potential of structured activity to influence how individuals experience and evaluate their physical capabilities. At the same time, repeated mastery experiences and successful participation may gradually increase confidence in physical abilities, encouraging more positive evaluations of the physical self (McIntyre et al., 2015). Rather than focusing solely on changes in physical appearance, structured physical activity may also shift attention toward what the body is capable of achieving, thereby promoting a more positive and functional perception of the self.

The multidimensional nature of physical self-concept may also help explain the positive pooled effect observed in the present review. Physical self-concept encompasses several related but distinct domains, including sport competence, physical condition, physical strength, body attractiveness, and physical self-worth (Fox and Corbin, 1989; Lindwall et al., 2011). Different physical activity programmes may therefore influence different dimensions of physical self-concept. For example, resistance training has been associated with improvements in perceived body attractiveness (Lubans et al., 2010), whereas individualized physical education programmes have been shown to enhance endurance- and strength-related self-concepts (Schmidt et al., 2013). Although intervention content may differ, structured physical activity appears to provide repeated opportunities for positive physical experiences that contribute to improvements across multiple dimensions of physical self-concept.

The present findings suggest that the potential benefits of physical activity interventions may extend beyond physical fitness. Structured physical activity provides children and adolescents with opportunities to experience competence, achievement, and personal improvement, all of which may contribute to more positive perceptions of the physical self. Therefore, intervention programmes should not only aim to increase physical activity participation but also deliberately incorporate activities that promote successful movement experiences and reinforce positive self-perceptions. Such an approach may maximize both the physical and psychological benefits of physical activity.

## 5 Limitations and future directions

First, considerable heterogeneity remained across the included studies. Although random-effects models together with subgroup and meta-regression analyses were used to explore potential sources of variability, a large proportion of the heterogeneity remained unexplained. Such variability is likely attributable to differences in intervention design, participant profiles, and the multidimensional nature of physical self-concept. In addition, sufficient information was unavailable to fully account for cluster- or site-level allocation in several studies, which may have affected the precision and weighting of their effect estimates. Although sensitivity analysis excluding these studies yielded a consistent direction of effect, the wider confidence interval indicated reduced precision and greater uncertainty in the effect estimate. Future studies should provide more detailed methodological and intervention information to facilitate more precise comparisons across studies.

Second, a range of validated instruments was used to evaluate physical self-concept across the included studies. Although all measured related constructs, differences in questionnaire structure and the specific dimensions assessed may partly explain the variability observed in the pooled effect estimates. Future research would benefit from greater consistency in outcome measurement.

Third, evidence from special populations was limited, and several subgroup analyses were based on only a few studies. Consequently, the available evidence remains insufficient to determine whether intervention effectiveness differs across specific populations or settings. Additional well-designed intervention studies involving diverse populations are needed to strengthen the evidence base.

Finally, only studies published in English were included, which may have introduced language bias and resulted in the omission of relevant evidence published in other languages. In addition, most included studies assessed physical self-concept immediately after the intervention, with relatively limited information regarding the long-term maintenance of intervention effects. Future research should therefore incorporate longer follow-up periods to determine whether improvements in physical self-concept are sustained after structured physical activity programmes have ended.

## 6 Conclusion

This systematic review and meta-analysis suggests that structured physical activity interventions may improve physical self-concept in children and adolescents. Positive effects were observed across a range of intervention types and participant characteristics, suggesting the potential benefits of physical activity for enhancing children's and adolescents' perceptions of their physical abilities and physical selves. These findings suggest that structured physical activity may be a useful strategy for promoting positive physical self-concept during childhood and adolescence.

## Data Availability

The original contributions presented in the study are included in the article/Supplementary material, further inquiries can be directed to the corresponding author/s.
