# Supplementary material for: How effective are physical activity interventions in improving physical self-concept among children and adolescents? A systematic review and meta-analysis
Source: Front Psychol. 2026 Oct 16;17:1963989. doi: 10.3389/fpsyg.2026.1963989 (PMC13719429; doi:10.3389/fpsyg.2026.1963989)
Supplement: Supplementary file 1 [file Supplementary_File_1.docx]

**Appendix 1：search strategy**

A systematic literature search was conducted across PubMed, Embase, Web of Science, the Cochrane Library, and EBSCO, covering all records published from database inception to June 10, 2026,

| **Database** | **Retrieval strategy** |
| --- | --- |
| **Cochranne （n=1193)** | ((child* OR adolescen* OR youth* OR teen* OR pediatric* OR paediatric* OR schoolchild* OR school-age* OR student*):ti,ab,kw AND ("physical activity" OR "physical activities" OR exercise* OR sport* OR training OR "physical education" OR exergam* OR "exercise intervention" OR "physical activity intervention" OR "sport intervention" OR "aerobic exercise" OR "resistance training" OR "strength training" OR "multicomponent exercise"):ti,ab,kw AND ("physical self-concept" OR "physical self concept" OR "physical self-concepts" OR "physical self concepts" OR "physical self-perception" OR "physical self perception" OR "physical self-perceptions" OR "physical self perceptions" OR "physical self-worth" OR "physical self worth" OR "physical self-description" OR "physical self description" OR PSDQ OR "PSDQ-S" OR "Physical Self-Description Questionnaire" OR "Physical Self Description Questionnaire" OR "Physical Self-Description Questionnaire-Short Form" OR PSPP OR "Physical Self-Perception Profile" OR "Physical Self Perception Profile" OR "CY-PSPP" OR "Children and Youth Physical Self-Perception Profile" OR "Children and Youth Physical Self Perception Profile" OR "Children's Physical Self-Perception Profile" OR "Children's Physical Self Perception Profile"):ti,ab,kw) |
| **Embase**  **(n=413)** | (((child*:ti,ab OR adolescen*:ti,ab OR youth*:ti,ab OR teen*:ti,ab OR pediatric*:ti,ab OR paediatric*:ti,ab OR schoolchild*:ti,ab OR school-age*:ti,ab OR student*:ti,ab) AND ("physical activity":ti,ab OR "physical activities":ti,ab OR exercise*:ti,ab OR sport*:ti,ab OR training:ti,ab OR "physical education":ti,ab OR exergam*:ti,ab OR "exercise intervention":ti,ab OR "physical activity intervention":ti,ab OR "sport intervention":ti,ab OR "aerobic exercise":ti,ab OR "resistance training":ti,ab OR "strength training":ti,ab OR "multicomponent exercise":ti,ab) AND ("physical self-concept":ti,ab OR "physical self concept":ti,ab OR "physical self-concepts":ti,ab OR "physical self concepts":ti,ab OR "physical self-perception":ti,ab OR "physical self perception":ti,ab OR "physical self-perceptions":ti,ab OR "physical self perceptions":ti,ab OR "physical self-worth":ti,ab OR "physical self worth":ti,ab OR "physical self-description":ti,ab OR "physical self description":ti,ab OR PSDQ:ti,ab OR "PSDQ-S":ti,ab OR "Physical Self-Description Questionnaire":ti,ab OR "Physical Self Description Questionnaire":ti,ab OR "Physical Self-Description Questionnaire-Short Form":ti,ab OR PSPP:ti,ab OR "Physical Self-Perception Profile":ti,ab OR "Physical Self Perception Profile":ti,ab OR "CY-PSPP":ti,ab OR "Children and Youth Physical Self-Perception Profile":ti,ab OR "Children and Youth Physical Self Perception Profile":ti,ab OR "Children's Physical Self-Perception Profile":ti,ab OR "Children's Physical Self Perception Profile":ti,ab)) |
| **Web of Science**  **(n=233)** | TS=((child* OR adolescen* OR youth* OR teen* OR pediatric* OR paediatric* OR schoolchild* OR school-age* OR student*) AND ("physical activity" OR "physical activities" OR exercise* OR sport* OR training OR "physical education" OR exergam* OR "exercise intervention" OR "physical activity intervention" OR "sport intervention" OR "aerobic exercise" OR "resistance training" OR "strength training" OR "multicomponent exercise") AND ("physical self-concept" OR "physical self concept" OR "physical self-concepts" OR "physical self concepts" OR "physical self-perception" OR "physical self perception" OR "physical self-perceptions" OR "physical self perceptions" OR "physical self-worth" OR "physical self worth" OR "physical self-description" OR "physical self description" OR PSDQ OR "PSDQ-S" OR "Physical Self-Description Questionnaire" OR "Physical Self Description Questionnaire" OR "Physical Self-Description Questionnaire-Short Form" OR PSPP OR "Physical Self-Perception Profile" OR "Physical Self Perception Profile" OR "CY-PSPP" OR "Children and Youth Physical Self-Perception Profile" OR "Children and Youth Physical Self Perception Profile" OR "Children's Physical Self-Perception Profile" OR "Children's Physical Self Perception Profile")) |
| **PubMed**  **(n=286)** | ((child*[Title/Abstract] OR adolescen*[Title/Abstract] OR youth*[Title/Abstract] OR teen*[Title/Abstract] OR pediatric*[Title/Abstract] OR paediatric*[Title/Abstract] OR schoolchild*[Title/Abstract] OR school-age*[Title/Abstract] OR student*[Title/Abstract]) AND ("physical activity"[Title/Abstract] OR "physical activities"[Title/Abstract] OR exercise*[Title/Abstract] OR sport*[Title/Abstract] OR training[Title/Abstract] OR "physical education"[Title/Abstract] OR exergam*[Title/Abstract] OR "exercise intervention"[Title/Abstract] OR "physical activity intervention"[Title/Abstract] OR "sport intervention"[Title/Abstract] OR "aerobic exercise"[Title/Abstract] OR "resistance training"[Title/Abstract] OR "strength training"[Title/Abstract] OR "multicomponent exercise"[Title/Abstract]) AND ("physical self-concept"[Title/Abstract] OR "physical self concept"[Title/Abstract] OR "physical self-concepts"[Title/Abstract] OR "physical self concepts"[Title/Abstract] OR "physical self-perception"[Title/Abstract] OR "physical self perception"[Title/Abstract] OR "physical self-perceptions"[Title/Abstract] OR "physical self perceptions"[Title/Abstract] OR "physical self-worth"[Title/Abstract] OR "physical self worth"[Title/Abstract] OR "physical self-description"[Title/Abstract] OR "physical self description"[Title/Abstract] OR PSDQ[Title/Abstract] OR "PSDQ-S"[Title/Abstract] OR "Physical Self-Description Questionnaire"[Title/Abstract] OR "Physical Self Description Questionnaire"[Title/Abstract] OR "Physical Self-Description Questionnaire–Short Form"[Title/Abstract] OR "Physical Self-Description Questionnaire-Short Form"[Title/Abstract] OR PSPP[Title/Abstract] OR "Physical Self-Perception Profile"[Title/Abstract] OR "Physical Self Perception Profile"[Title/Abstract] OR "CY-PSPP"[Title/Abstract] OR "Children and Youth Physical Self-Perception Profile"[Title/Abstract] OR "Children and Youth Physical Self Perception Profile"[Title/Abstract] OR "Children's Physical Self-Perception Profile"[Title/Abstract] OR "Children's Physical Self Perception Profile"[Title/Abstract])) |
| **SPORTDiscus (via EBSCOhost)**  **(n=330)** | ((TI(child* OR adolescen* OR youth* OR teen* OR pediatric* OR paediatric* OR schoolchild* OR school-age* OR student*) OR AB(child* OR adolescen* OR youth* OR teen* OR pediatric* OR paediatric* OR schoolchild* OR school-age* OR student*)) AND (TI("physical activity" OR "physical activities" OR exercise* OR sport* OR training OR "physical education" OR exergam* OR "exercise intervention" OR "physical activity intervention" OR "sport intervention" OR "aerobic exercise" OR "resistance training" OR "strength training" OR "multicomponent exercise") OR AB("physical activity" OR "physical activities" OR exercise* OR sport* OR training OR "physical education" OR exergam* OR "exercise intervention" OR "physical activity intervention" OR "sport intervention" OR "aerobic exercise" OR "resistance training" OR "strength training" OR "multicomponent exercise")) AND (TI("physical self-concept" OR "physical self concept" OR "physical self-concepts" OR "physical self concepts" OR "physical self-perception" OR "physical self perception" OR "physical self-perceptions" OR "physical self perceptions" OR "physical self-worth" OR "physical self worth" OR "physical self-description" OR "physical self description" OR PSDQ OR "PSDQ-S" OR "Physical Self-Description Questionnaire" OR "Physical Self Description Questionnaire" OR "Physical Self-Description Questionnaire-Short Form" OR PSPP OR "Physical Self-Perception Profile" OR "Physical Self Perception Profile" OR "CY-PSPP" OR "Children and Youth Physical Self-Perception Profile" OR "Children and Youth Physical Self Perception Profile" OR "Children's Physical Self-Perception Profile" OR "Children's Physical Self Perception Profile") OR AB("physical self-concept" OR "physical self concept" OR "physical self-concepts" OR "physical self concepts" OR "physical self-perception" OR "physical self perception" OR "physical self-perceptions" OR "physical self perceptions" OR "physical self-worth" OR "physical self worth" OR "physical self-description" OR "physical self description" OR PSDQ OR "PSDQ-S" OR "Physical Self-Description Questionnaire" OR "Physical Self Description Questionnaire" OR "Physical Self-Description Questionnaire-Short Form" OR PSPP OR "Physical Self-Perception Profile" OR "Physical Self Perception Profile" OR "CY-PSPP" OR "Children and Youth Physical Self-Perception Profile" OR "Children and Youth Physical Self Perception Profile" OR "Children's Physical Self-Perception Profile" OR "Children's Physical Self Perception Profile"))) |
